# Selective migration of neuralized embryonic stem cells to stem cell factor and media conditioned by glioma cell lines

**DOI:** 10.1186/1475-2867-6-1

**Published:** 2006-01-25

**Authors:** Peter Serfozo, Maggie S Schlarman, Chris Pierret, Bernard L Maria, Mark D Kirk

**Affiliations:** 1 Division of Biological Sciences, 114 Lefevre Hall, University of Missouri, Columbia MO 65211; 2 Charles P. Darby Children's Research Institute, Medical University of South Carolina, 135 Rutledge Ave., Charleston, SC 29425

## Abstract

**Background:**

Pluripotent mouse embryonic stem (ES) cells can be induced *in vitro *to become neural progenitors. Upon transplantation, neural progenitors migrate toward areas of damage and inflammation in the CNS. We tested whether undifferentiated and neuralized mouse ES cells migrate toward media conditioned by glioma cell lines (C6, U87 & N1321) or Stem Cell Factor (SCF).

**Results:**

Cell migration assays revealed selective migration by neuralized ES cells to conditioned media as well as to synthetic SCF. Migration of undifferentiated ES cells was extensive, but not significantly different from that of controls (Unconditioned Medium). RT-PCR analysis revealed that all the three tumor cell lines tested synthesized SCF and that both undifferentiated and neuralized ES cells expressed *c-kit*, the receptor for SCF.

**Conclusion:**

Our results demonstrate that undifferentiated ES cells are highly mobile and that neural progenitors derived from ES cells are selectively attracted toward factors produced by gliomas. Given that the glioma cell lines synthesize SCF, SCF may be one of several factors that contribute to the selective migration observed.

## Background

Embryonic stem (ES) cells possess the capacity for unlimited self renewal and can be induced *in vitro *to become neural precursors with the potential for therapeutic treatment of nervous system disorders [1-5]. Neural stem cells (NSCs) are mobile [6], are attracted to regions of brain injury and can migrate great distances to reach a site of neural damage [7-10]. In addition, through unknown mechanisms, they exhibit tropism to brain tumors, including glioma cells that have left the main tumor mass and have infiltrated adjacent brain parenchyma [6,8,11,12]. *In vitro *migration assays confirm the ability of isolated NSCs, including those derived from mouse embryonic stem cells [13], to migrate toward factors produced by glioma cells [8,14].

Recent studies suggest that stem cell factor (SCF) and stromal cell-derived factor 1α (SDF1α) act as chemoattractants, capable of inducing neural stem cell migration into regions of brain injury/inflammation. For example, Sun et al. [15] report that in normal mouse brains, endogenous NSCs are attracted to regions where recombinant SCF has been introduced, SCF elicits selective migration of neural stem/progenitor cells *in vitro*, and after a freezing brain injury SCF is up-regulated in neurons at the site of injury. Also, Imitola et al. [16] found in a mouse stroke model that SDF1α synthesis by astrocytes and endothelial cells is increased at the site of injury and that exogenous human NSCs migrate to sites of injury from as far as the contralateral hemisphere to intermingle with SDF1α-expressing cells. These studies suggest that cytokines, such as SCF and SDF1α may be involved in attracting stem cells to regions of injury and inflammation [17].

Since brain tumors can also attract stem cells, perhaps their mechanism of attraction is similar to that of injury and inflammation. Support for this comes from reports of the expression of SCF by certain glioma cell lines [18,19] and expression of *c-kit*, the tyrosine kinase receptor for SCF ligand, by neural stem/progenitor cells [15,20]. Clearly, it is important to characterize the reactions of stem cells to gliomas, including whether they display the capacity for selective attraction to tumor cells. In the present study, we performed *in vitro *migration assays to compare the behavior of undifferentiated and neuralized mouse ES cells toward the human glioma lines U87 and N1321, rat glioma line C6 and SCF. In addition, we tested for expression of SCF by the tumor lines and of *c-kit *by the ES cells.

## Results

### Neuralized ES cells selectively migrate to factor(s) produced by glioma cell lines

We used *in vitro *migration assays to test whether undifferentiated or neuralized ES cells displayed selective migration toward factors produced by rat glioma cell line C6 or human glioma cell lines U87 and N1231. The migration experiments consisted of placing either undifferentiated or neuralized ES cells (at Day 4 or Day 8 of neural induction) in the top well and a selected tumor cell line or media conditioned by a tumor cell line in the bottom well. If the glioma cell lines produced attractants, then they should cause significantly more stem cells to migrate from the top well, through the porous membrane toward the bottom well when compared to Unconditioned Medium.

**Figure 1:**
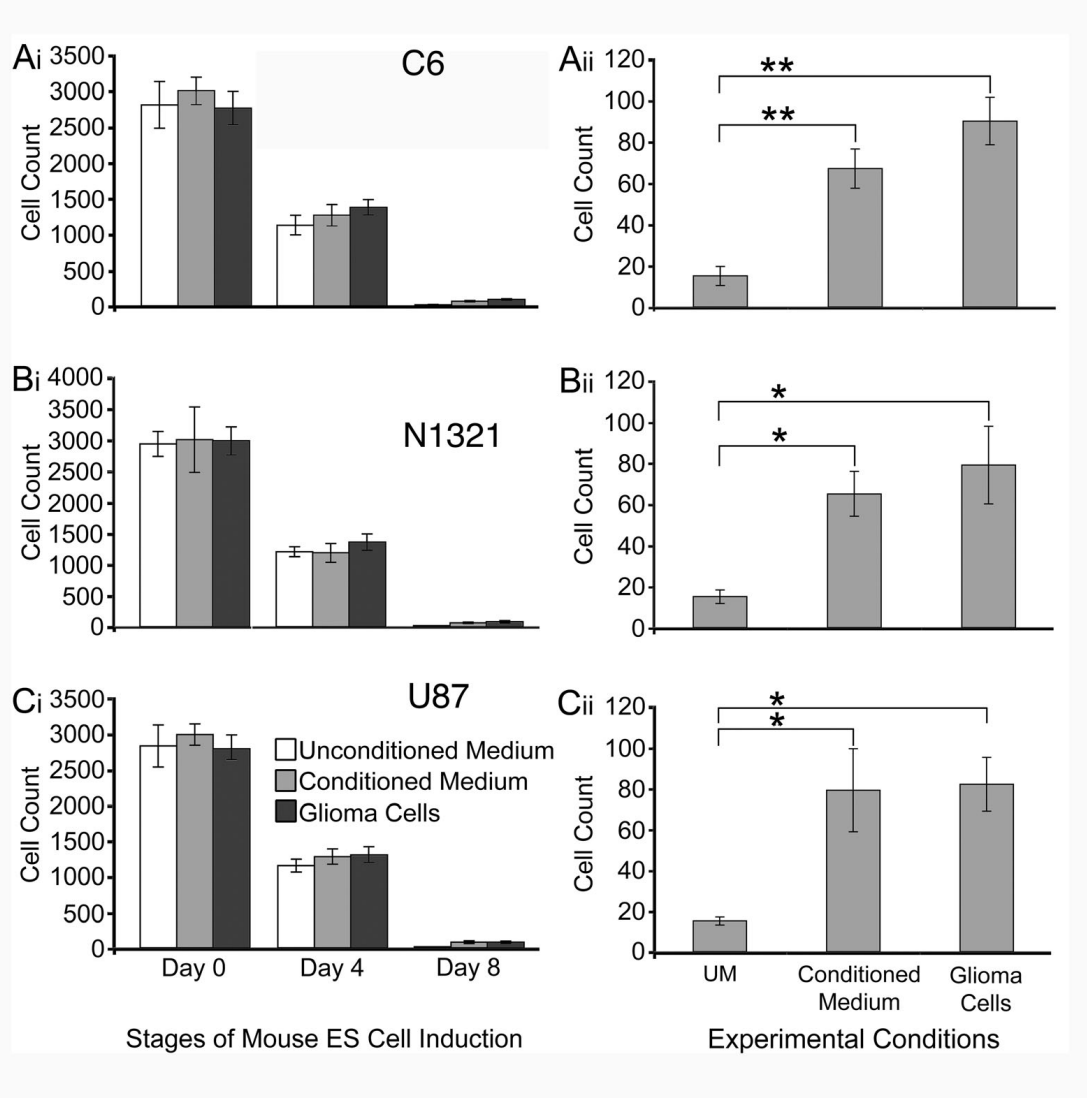
Undifferentiated ES cells exhibit extreme mobility and neuralized ES cells exhibit selective migration toward Glioma Cells and Conditioned Medium. **A-C**, Cell counts were made after a 4 hours incubation with either Unconditioned Medium (UM; DMEM and FBS alone), Conditioned Medium or Glioma Cells in the bottom well, for all three glioma cell lines, C6, N1321, and U87 (as indicated). On Day 0 (undifferentiated ES cells) and on Day 4, large numbers of stem cells migrated toward the bottom well with no significant differences in cell counts, regardless of the content of the lower well (Ai-Ci). However, neuralized ES cells obtained at Day 8 of the induction protocol exhibited selective migration when Conditioned Medium or Glioma cells were placed in the bottom well for all three glioma cell lines, C6, N1321, and U87, respectively (Aii-Cii). At Day 8, significance was established at p < 0.05 (*) or p < 0.01 (**) using One-Way ANOVA and a post hoc Dunnet Test.

Large numbers of undifferentiated ES cells (Day 0 of induction) migrated toward the bottom chambers with no significant differences observed in response to the contents of the bottom well (Fig. 1Ai,Bi,Ci). By Day 4 of neural induction, there also were no significant differences in cell counts among the three experimental conditions (Fig. 1). Note that for all experimental conditions, migration on Day 4 of neural induction was significantly (p < 0.01) lower than on Day 0 (i.e., comparisons were made for cell counts in response to Unconditioned Medium and for combined cell counts of Conditioned Medium and Glioma Cells). These results suggested a decreased mobility of the ES cells as they began to differentiate.

Migration on Day 8 of neural induction indicated a continued and significant decrease in mobility, when compared to Day 0 or Day 4 of induction (Fig. 1). Importantly however, the neuralized ES cells on Day 8 of induction showed selective migration; that is, significantly more cells migrated toward Conditioned Medium and Tumor Cells for all three glioma cell lines, when compared to Unconditioned Medium (Fig. 1Aii,Bii,Cii). These results suggested that all three glioma cell lines secrete a factor(s) that acts on the neuralized ES cells as an attractant.

### Selective migration of neualized ES cells to SCF

**Figure 2:**
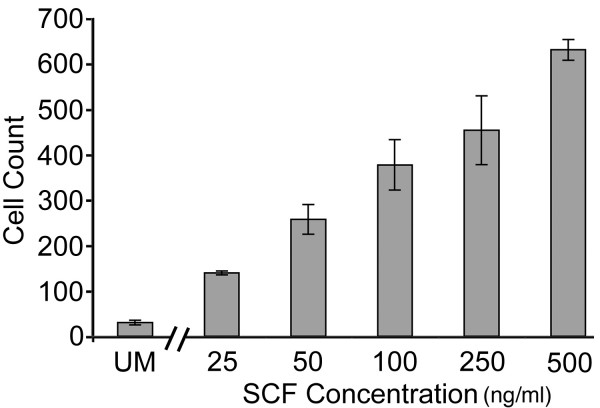
Stem Cell Factor (SCF) elicited a concentration-dependent migration of neuralized ES cells (Day 8). At concentrations above 50 ng/ml of recombinant SCF, significantly more cells migrated towards the bottom well when compared to Unconditioned Medium (p < 0.001; Newman-Keuls Multiple Comparisons Test).

One factor that is upregulated in regions of CNS injury and inflammation and attracts migratory neural stem cells is Stem Cell Factor or SCF [15]. Cells isolated on Day 8 of neural induction were highly attracted to SCF (Fig. 2). The numbers of cells that migrated through the membrane to SCF did so in an apparent dose-dependent manner. At concentrations of 50 ng/ml or higher their migration was significantly (p < 0.001) greater than for Unconditioned Medium. No significant differences in numbers of cells were observed in response to recombinant SCF when the migration experiments were performed on Day 0 and Day 4 of induction (data not shown).

### Glioma cell lines express SCF and mouse ES cells express *c-kit*

**Figure 3:**
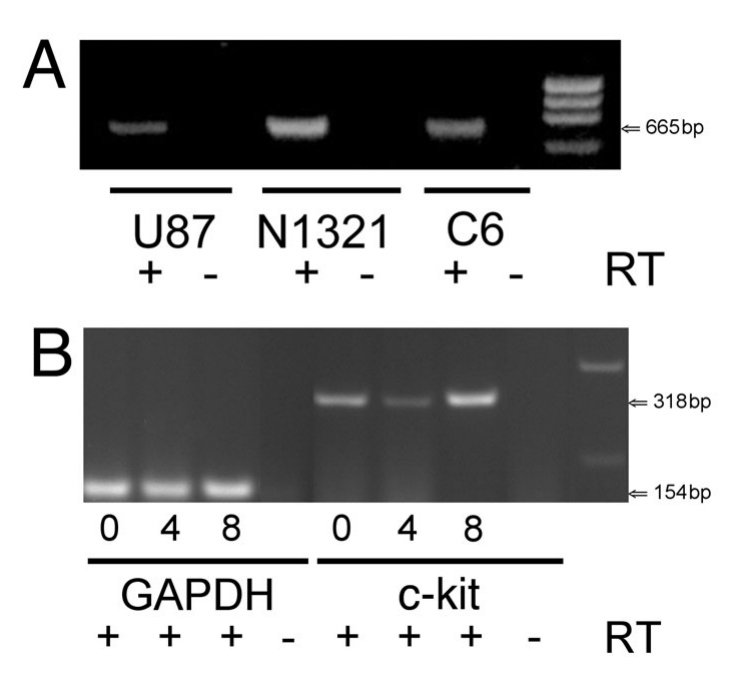
All three glioma cell lines express the secreted version of SCF **(A)**, and the undifferentiated and neurzalized ES cells express *c-kit*, the tyrosine kinase receptor for SCF. The ES cells expressed *c-kit *at 0, 4 and 8 Days of the induction protocol **(B)**. **A**, Primers specific for the splice variant of SCF that contains exon 6, known to produce secreted SCF, were used in RT-PCR experiments on glioma cell lines N1321, U87, and C6. The predicted 665 bp product is produced by all three cell lines. **B**, RT-PCR and primers specific for mouse *c-kit *demonstrated that undifferentiated ES cells and Day 4 and Day 8 neuralized ES cells express *c-kit *(318 bp PCR product). Primers specific for mouse GAPDH (154 bp PCR product) were used as loading controls and are shown for the corresponding days of induction (taken from the same gel as for *c-kit *expression). As a negative control reverse transcriptase (RT) was left out of the reaction mixture (- RT). No RT controls in **B **were pooled samples taken from cells at 0, 4 and 8 Days of induction. In the right column of **A **and **B **is shown a low DNA mass ladder (Invitrogen, Cat. # 10068-013).

Given that SCF elicited migration of neural progenitors derived from ES cells, we tested whether the three glioma cell lines expressed SCF. Splice variants of human SCF give rise to two forms of SCF, secreted and membrane bound. SCF transcripts containing exon 6 give rise to protein with an extracellular cleavage site that leads to production of soluble SCF [18]. We designed PCR primers that would result in a 665 bp product if the exon 6 coding region is present. Using RT-PCR we observed expression of a 665 bp mRNA in all three glioma cell lines (Fig. 3A), indicating synthesis of the secreted form of SCF.

Using RT-PCR and primers specific for mouse *c-kit *(318 bp PCR product), we confirmed that undifferentiated ES cells and Day 4 and Day 8 induced cells expressed *c-kit *(Fig. 3B). Expression of gylceraldehyde-3-phosphate dehydrogenase (GAPDH) was used as a house-keeping control.

## Discussion

Our results show that neural progenitors derived from mouse ES cells migrate toward factor(s) secreted by glioma cell lines U87, N1321 and C6. We show that these glioma lines can synthesize SCF and that recombinant SCF elicits migration by neuralized ES cells in an apparent dose-dependent manner. This selective migration is consistent with the expression of *c-kit*, the receptor for SCF, by the ES cell-derived neural progenitors.

Prior to this study, little was known about the migratory properties of undifferentiated ES cells [21-23]. In contrast to neuralized ES cells, we found that *in vitro *undifferentiated ES cells are highly migratory in all conditions. The highly migratory nature of undifferentiated ES cells may contribute to their unique roles in early embryonic development, such as migration events leading to gastrulation.

The migration of neuralized ES cells towards conditioned medium *in vitro *may not be selective for SCF because the glioma cell lines likely produce other attractant factors. However, the production of SCF by glioma cells and expression of *c-kit *by neural precursors suggests that SCF could mediate selective migration towards gliomas *in vivo*.

Our initial migration studies led to the question: what factors are secreted by the gliomas that act on neuralized ES cells to elicit selective migration? Malignant gliomas secrete a wide variety of factors, associated with their proliferative and invasive programs, including cytokines, interleukins and growth factors, such as TGF-β1 [24-26], and matrix metalloproteinases [27]. The cytokine SCF elicits selective migration of brain-derived neural stem/progenitor cells *in vitro *[28] and is up-regulated in response to brain injury [15]. We confirmed expression of SCF by the human glioma cell line, U87 [19]. In addition, we found that the human glioma cell line, N1321, and the rat glioma cell line, C6, both express SCF, and the version of SCF expressed by these cell lines contains exon 6. Exon 6 is present in the splice variant of SCF from which soluble SCF is produced [18]. It is also known that many types of stem cells express the receptor for SCF, *c-kit *[20,29,30]. We confirmed expression of *c-kit *by undifferentiated mouse ES cells [31] and found that neuralized ES cells also express *c-kit*. The latter result is consistent with expression of *c-kit *by neural stem cells as well as with the attractant and survival effects of SCF on neural stem cells [20,28]. It is possible that expression of *c-kit *in Day 8 embryoid bodies (Fig. 3B) is due to the presence of neural progenitors [1,3]. While our data suggest that SCF may be involved in eliciting selective migration by neuralized ES cells, glioma cell lines likely produce other attractants. In the future, it will be important to test the contribution of SCF (if any) to selective migration documented here by adding antibodies to SCF to the top well, to potentially block the actions of SCF diffusing from the bottom well.

Neural stem cells demonstrate remarkable tropism to factors produced by gliomas *in vivo *[8,12,16,32]. Importantly, they can be genetically modified to express therapeutic transgenes. These transgenes can encode oncolytic agents, apoptosis-inducing factors, interleukins, factors that inhibit angiogenesis and factors that sensitize tumor cells to traditional treatments for gliomas, such as chemotherapy and radiation [11,32,33]. Recent results show that transplanted neural precursors can improve survival and reduce tumor volume in rodent models with introduced glioblastomas [8,11,34]. In fact, transplanted and endogenous neural precursors as well as bone marrow-derived mesenchymal cells [35,36], may enhance survival after induction of glioblastomas in rodent models [37]. The 4-/4+ induction protocol used here produces a heterogeneous mixture of neural cells within the EBs. It will be important to test whether neural progenitors, or more mature neural cells present in Day 8 EBs, contribute to the cell population that is selectively attracted to SCF and to medium conditioned by the glioma cell lines.

## Conclusion

Because of their highly invasive nature, most gliomas are not eradicated by traditional therapies, and consequently are often fatal [38]. Clearly, therapies using stem cells as vectors to deliver anti-tumor agents offer a promising direction for new treatment strategies [13,17,34,39]. In addition, neural stem/progenitor cells derived from ES cells could help rebuild regions of the CNS damaged by glioma or its treatment (surgery, radiation therapy and/or chemotherapy).

## Methods

### ES Cell cultures and neural induction

The B5 mouse ES cell line [3,40] was used for all experiments. The ES cells were grown in embryonic stem cell growth medium (ESGM) (as described previously [3]) for 2 days on gelatin-coated flasks until 70% confluent. The cells were then dissociated at 37°C for 5 minutes (0.25% Trypsin with 1 mM EDTA), passed into 4 gelatin-coated flasks and incubated for an additional 2 days (37°C, 5% C0_2_). Then dissociated ES cells were transferred to uncoated petri plates and induced (i.e., neuralized) to become neural precursors as free floating embryoid bodies (EBs), using a retinoic acid induction protocol developed by Gottlieb and colleagues [1,3]. Prior to migration assays, EBs were grown for 4 days (Day 4) in embryonic stem cell induction medium (ESIM = ESGM without β-mercaptoethanol and Leukemia Inhibitory Factor) or for an additional 4 days (Day 8) in ESIM plus all-trans retinoic acid (500 nM).

As we reported previously [3], post-induction EBs obtained using B5 ES cells contain a majority of cells that express the neural precursor marker Nestin, but these EBs have substantial numbers of cells that label for neuronal markers, such as β-III Tubulin and Neurofilament-M. Therefore, cells used for the migration assays represent a heterogeneous population, consisting primarily of neural progenitors and/or neural-like cells. After induction, EBs were treated with 0.25% Trypsin with 1 mM EDTA, dissociated mechanically to a single cell suspension and 25,000 cells were added to each well of the top chamber.

### Tumor cell lines

Human glioma cell lines N1321 and U87 and rat glioma cell line C6 were grown in DMEM and 10% fetal bovine serum supplemented with 100 U/ml Penicillin, 100 μg/ml Streptomycin [41] for 2 days in Tissue Culture BD Falcon Flasks (Fisher; Cat. # 13-680-65). The U87 and C6 cell lines were dissociated at 37°C for 2.5 minutes using 0.25% Trypsin with 1 mM EDTA. The N1321 cell line was also dissociated for 2 minutes but using 0.05% Trypsin with 1 mM EDTA. Cells were then passaged and incubated for an additional 2 days (37°C, 5% C0_2_). All cells (ES and glioma cells) were tested for viability using Trypan blue stain and live cells were counted using a hemocytometer.

### Migration assays and statistical analyses

Cell migration tests were performed using the Neuro Probe Standard 48 Well Chemotaxis Chamber (Cat. # AP48). The lower well was filled with either Unconditioned Medium, medium conditioned by one of the glioma cell lines (i.e., Conditioned Medium) or glioma cells at a density of 50,000 cells per 30 μl. Conditioned Medium was obtained by collecting medium from glioma cell cultures after 2 days of incubation. In migration assays involving SCF, recombinant SCF was added in the bottom well at selected concentrations. A porous polycarbonate membrane (8 μm pores) was coated with entactin-collagen IV-laminin (Upstate Biotechnologies, Cat. # 08–110) and placed on top of the bottom chamber.

Wells of the top chamber were filled with a suspension of stem/progenitor cells in DMEM at a density of 25,000 cells per 50 μl with cells at three different stages of differentiation as follows: undifferentiated embryonic stem cells (Day 0) or stem cells at Day 4 or Day 8 of the induction protocol. Table 1 lists the components found in the top and bottom wells for experiments described here. Negative controls for cellular migration through the membrane included: 1- DMEM alone in the top well and glioma cells in the bottom well, and 2- DMEM alone in the top well and Unconditioned Medium (DMEM and FBS) in the bottom well (Table 1). The entire chamber was placed in an incubator (37°C, 5% C02) for 4 hours. The chamber was separated and cells on the polycarbonate membrane were fixed in methanol for 10 minutes and washed in PBS (4X for 2 minutes each). The fixed membrane was stained for 3 hours with Hoechst 33358 nuclear stain at 50 μg/ml to identify cells that had migrated through the membrane pores. After staining, the membrane was washed with PBS (5X for 2 minutes each). The membrane was then placed on a slide for subsequent epifluorescence microscopy.

**Table 1:** Contents of Top and Bottom Wells for Migration Experiments

Stages of ES Cell Induction and Experimental Condition		Days 0, 4, 8 and Glioma Cells	Days 0, 4, 8 and Conditioned Medium	Day 8 and rSCF	Days 0, 4, 8 and Negative Control 1*	Days 0, 4, 8 and Negative Control 2*
Top Well						
	DMEM	X	X	X	X	X
	Stem Cells	X	X	X		

Bottom Well						
	DMEM	X	X	X	X	X
	FBS	X	X	X	X	X
	Glioma Cells	X			X	
	Factors Secreted by Tumor Cells	X	X		X	
	rSCF			X		

* See Methods

Unconditioned Medium = DMEM and FBS

Abbreviations: rSCF, recombinant Stem Cell Factor; DMEM, Dulbecco's modified eagle medium; FBS, fetal bovine serum

Images were captured digitally using a Leica stereoscope, Model MZFLIII, equipped with a CCD camera. Images were saved as TIFF files using MagnaFire (Ver. 2.1c) and analyzed using NIH Image (Ver. 1.62). The total numbers of cells that migrated through the pores of the membrane were counted. All experimental conditions were replicated a minimum of three times, and all experiments were performed at least three times.

Statistical comparisons of cell counts were made using One-Way ANOVA and post hoc Dunnet Test and/or Newman-Keuls Multiple Comparisons Test. Significance is given at p < 0.05 level, unless otherwise noted.

### RT-PCR

Total RNA isolation was performed using GenElute according to manufacturer's instructions (Sigma-Aldrich, Cat# RTN 10). The 50 μl isolate was treated with 5 μl DNase1 and 5 μl Reaction Buffer for 10 minutes at 37°C. Then, 5 μl stop buffer was added and the mixture held at 70°C for 10 minutes. The cDNA was created with Marligen Biosciences Inc. First-Strand cDNA Synthesis System (Cat#11801-100) as directed by the manufacturer. PCR was run using Eppendorf's HotMasterMix (2.5X) (Cat# 954140181), 200 uM primers and 7 μl of cDNA template. Controls lacking Reverse Transcriptase were included. In Figure 3B, no RT controls were pooled prior to PCR. The SCF primers used were 5'-AAGGGATCTGCAGGAATCGTGTGA-3' (forward) and 5'-TGCCCTTGTAAGACTTGG CTGTCT-3' (reverse). The parameters were 32 cycles at 94°C for 1 min, 55°C for 1 min, and 72°C for 1 min, with final elongation at 72°C for 10 minutes. The mouse *c-kit *primers used were 5'-CCATGTGGCTAAAGATGAAC-3' (upstream) and 5'-CTGCTGGTGCTCGGGTTTG-3' (downstream) [15]. The GAPDH primers used were 5'- TGATGGGTGTGAACCACGAGAA -3' (upstream) and 5'- AGTGATGGCATGGACTGTGGTCAT-3' (downstream). The parameters for *c-kit *and GAPDH amplification were as follows: 30 cycles at 94°C for 30 sec, 54°C for 45 sec, 69°C for 45 sec, with final elongation at 69°C for 10 minutes.

## Abbreviations

SCF, Stem Cell Factor; NSCs, neural stem cells; ES, embryonic stem; SDF1α, stromal cell-derived factor 1α; TGF-β1, transforming growth factor-beta one; PT- PCR, reverse transcriptase-polymerase chain reaction; ESGM, embryonic stem cell growth medium; ESIM, embryonic stem cell induction medium; EBs, embryoid bodies; GAPDH, Gylceraldehyde-3-phosphate dehydrogenase.

## Competing interests

The author(s) declare that they have no competing interests.

## Authors' contributions

PS & MS carried out the cell cultures and migration assays. CP & MS participated in the RT-PCR. BM & MK conceived of the study and participated in its design and coordination. MS & MK drafted the manuscript. All authors read and approved the final manuscript.

## References

[B1] Bain G, Kitchens D, Yao M, Huettner JE, Gottlieb DI (1995). Embryonic stem cells express neuronal properties in vitro. Dev Biol.

[B2] Lang KJ, Rathjen J, Vassilieva S, Rathjen PD (2004). Differentiation of embryonic stem cells to a neural fate: a route to re-building the nervous system?. J Neurosci Res.

[B3] Meyer JS, Katz ML, Maruniak JA, Kirk MD (2004). Neural differentiation of mouse embryonic stem cells in vitro and after transplantation into eyes of mutant mice with rapid retinal degeneration. Brain Res.

[B4] Okada Y, Shimazaki T, Sobue G, Okano H (2004). Retinoic-acid-concentration-dependent acquisition of neural cell identity during in vitro differentiation of mouse embryonic stem cells. Dev Biol.

[B5] Park SH, Kook MC, Kim EY, Park S, Lim JH (2004). Ultrastructure of human embryonic stem cells and spontaneous and retinoic acid-induced differentiating cells. Ultrastruct Pathol.

[B6] Fricker RA, Carpenter MK, Winkler C, Greco C, Gates MA, Bjorklund A (1999). Site-specific migration and neuronal differentiation of human neural progenitor cells after transplantation in the adult rat brain. J Neurosci.

[B7] Aarum J, Sandberg K, Haeberlein SL, Persson MA (2003). Migration and differentiation of neural precursor cells can be directed by microglia. Proc Natl Acad Sci U S A.

[B8] Aboody KS, Brown A, Rainov NG, Bower KA, Liu S, Yang W, Small JE, Herrlinger U, Ourednik V, Black PM, Breakefield XO, Snyder EY (2000). Neural stem cells display extensive tropism for pathology in adult brain: evidence from intracranial gliomas. Proc Natl Acad Sci U S A.

[B9] Haas S, Weidner N, Winkler J (2005). Adult stem cell therapy in stroke. Curr Opin Neurol.

[B10] Yip S, Aboody KS, Burns M, Imitola J, Boockvar JA, Allport J, Park KI, Teng YD, Lachyankar M, McIntosh T, O'Rourke DM, Khoury S, Weissleder R, Black PM, Weiss W, Snyder EY (2003). Neural stem cell biology may be well suited for improving brain tumor therapies. Cancer J.

[B11] Ehtesham M, Kabos P, Kabosova A, Neuman T, Black KL, Yu JS (2002). The use of interleukin 12-secreting neural stem cells for the treatment of intracranial glioma. Cancer Res.

[B12] Tang Y, Shah K, Messerli SM, Snyder E, Breakefield X, Weissleder R (2003). In vivo tracking of neural progenitor cell migration to glioblastomas. Hum Gene Ther.

[B13] Arnhold S, Hilgers M, Lenartz D, Semkova I, Kochanek S, Voges J, Andressen C, Addicks K (2003). Neural precursor cells as carriers for a gene therapeutical approach in tumor therapy. Cell Transplant.

[B14] Benedetti S, Pirola B, Pollo B, Magrassi L, Bruzzone MG, Rigamonti D, Galli R, Selleri S, Di Meco F, De Fraja C, Vescovi A, Cattaneo E, Finocchiaro G (2000). Gene therapy of experimental brain tumors using neural progenitor cells. Nat Med.

[B15] Sun L, Lee J, Fine HA (2004). Neuronally expressed stem cell factor induces neural stem cell migration to areas of brain injury. J Clin Invest.

[B16] Imitola J, Raddassi K, Park KI, Mueller FJ, Nieto M, Teng YD, Frenkel D, Li J, Sidman RL, Walsh CA, Snyder EY, Khoury SJ (2004). Directed migration of neural stem cells to sites of CNS injury by the stromal cell-derived factor 1alpha/CXC chemokine receptor 4 pathway. Proc Natl Acad Sci U S A.

[B17] Muller FJ, Snyder E, Loring FF (2006). Gene therapy: can neural stem cells deliver?. Nature Neurosci Rev.

[B18] Hamel W, Westphal M (1997). The road less travelled: c-kit and stem cell factor. J Neurooncol.

[B19] Stanulla M, Welte K, Hadam MR, Pietsch T (1995). Coexpression of stem cell factor and its receptor c-Kit in human malignant glioma cell lines. Acta Neuropathol (Berl).

[B20] Das AV, James J, Zhao X, Rahnenfuhrer J, Ahmad I (2004). Identification of c-Kit receptor as a regulator of adult neural stem cells in the mammalian eye: interactions with Notch signaling. Dev Biol.

[B21] Rippon HJ, Bishop AE (2004). Embryonic stem cells. Cell Prolif.

[B22] Rossant J (2001). Stem cells from the mammalian blastocyst. Stem Cells.

[B23] Smith AG (2001). Embryo-derived stem cells: of mice and men. Annu Rev Cell Dev Biol.

[B24] Brat DJ, Bellail AC, Van Meir EG (2005). The role of interleukin-8 and its receptors in gliomagenesis and tumoral angiogenesis. Neuro-oncol.

[B25] Mentlein R, Held-Feindt J (2002). Pleiotrophin, an angiogenic and mitogenic growth factor, is expressed in human gliomas. J Neurochem.

[B26] Teicher BA (2001). Malignant cells, directors of the malignant process: role of transforming growth factor-beta. Cancer Metastasis Rev.

[B27] Nagashima G, Suzuki R, Asai J, Fujimoto T (2002). Immunohistochemical analysis of reactive astrocytes around glioblastoma: an immunohistochemical study of postmortem glioblastoma cases. Clin Neurol Neurosurg.

[B28] Erlandsson A, Larsson J, Forsberg-Nilsson K (2004). Stem cell factor is a chemoattractant and a survival factor for CNS stem cells. Exp Cell Res.

[B29] Tran PB, Ren D, Veldhouse TJ, Miller RJ (2004). Chemokine receptors are expressed widely by embryonic and adult neural progenitor cells. J Neurosci Res.

[B30] Zhang SC, Fedoroff S (1999). Expression of stem cell factor and c-kit receptor in neural cells after brain injury. Acta Neuropathol (Berl).

[B31] Palmqvist L, Glover CH, Hsu L, Lu M, Bossen B, Piret JM, Humphries RK, Helgason CD (2005). Correlation of murine embryonic stem cell gene expression profiles with functional measures of pluripotency. Stem Cells.

[B32] Burns MJ, Weiss W (2003). Targeted therapy of brain tumors utilizing neural stem and progenitor cells. Front Biosci.

[B33] Hanna NN, Hallahan DE, Wayne JD, Weischselbaum RR (1996). Modification of the Radiation Response by the Administration of Exogenous Genes. Semin Radiat Oncol.

[B34] Shah K, Bureau E, Kim DE, Yang K, Tang Y, Weissleder R, Breakefield XO (2005). Glioma therapy and real-time imaging of neural precursor cell migration and tumor regression. Ann Neurol.

[B35] Abrey LE, Rosenblum MK, Papadopoulos E, Childs BH, Finlay JL (1999). High dose chemotherapy with autologous stem cell rescue in adults with malignant primary brain tumors. J Neurooncol.

[B36] Nakamizo A, Marini F, Amano T, Khan A, Studeny M, Gumin J, Chen J, Hentschel S, Vecil G, Dembinski J, Andreeff M, Lang FF (2005). Human bone marrow-derived mesenchymal stem cells in the treatment of gliomas. Cancer Res.

[B37] Glass R, Synowitz M, Kronenberg G, Walzlein JH, Markovic DS, Wang LP, Gast D, Kiwit J, Kempermann G, Kettenmann H (2005). Glioblastoma-induced attraction of endogenous neural precursor cells is associated with improved survival. J Neurosci.

[B38] Holland EC (2001). Brain tumor animal models: importance and progress. Curr Opin Oncol.

[B39] Studeny M, Marini FC, Dembinski JL, Zompetta C, Cabreira-Hansen M, Bekele BN, Champlin RE, Andreeff M (2004). Mesenchymal stem cells: potential precursors for tumor stroma and targeted-delivery vehicles for anticancer agents. J Natl Cancer Inst.

[B40] Hadjantonakis AK, Gertsenstein M, Ikawa M, Okabe M, Nagy A (1998). Generating green fluorescent mice by germline transmission of green fluorescent ES cells. Mech Dev.

[B41] Muir D, Johnson J, Rojiani M, Inglis BA, Rojiani A, Maria BL (1996). Assessment of laminin-mediated glioma invasion in vitro and by glioma tumors engrafted within rat spinal cord. J Neurooncol.

